# Supplementary material for: HIVprotI: an integrated web based platform for prediction and design of HIV proteins inhibitors
Source: J Cheminform. 2018 Mar 9;10:12. doi: 10.1186/s13321-018-0266-y (PMC5845081; doi:10.1186/s13321-018-0266-y)
Supplement: Supplementary file 2 — Additional file 2. Source code of HIVProtI web server. [file 13321_2018_266_MOESM2_ESM.zip › HIVprotI_Source-code/hivprotI/weka-3-6-6/doc/weka/associations/RuleItem.html]

RuleItem


---


|  |  |  |
| --- | --- | --- |
| **Overview** \| **Package** \| **Class** \| **Tree** \| **Deprecated** \| **Index** \| **Help** \| **Weka's home** | |  |
| **PREV CLASS**   **NEXT CLASS** | **FRAMES**    **NO FRAMES**     **All Classes** |  |
| SUMMARY: NESTED \| FIELD \| CONSTR \| METHOD | DETAIL: FIELD \| CONSTR \| METHOD |  |


---


## weka.associations Class RuleItem

```
java.lang.Object
  weka.associations.RuleItem
```

**All Implemented Interfaces:**: java.io.Serializable, java.lang.Comparable, RevisionHandler

---

``` public class RuleItem extends java.lang.Object implements java.lang.Comparable, java.io.Serializable, RevisionHandler ```

Class for storing an (class) association rule.
The premise and the consequence are stored each as separate item sets.
For every rule their expected predictive accuracy and the time of generation is stored.
These two measures allow to introduce a sort order for rules.

**Version:**
:   $Revision: 1.5 $

**Author:**
:   Stefan Mutter

**See Also:**: Serialized Form

---

**Constructor Summary**

`RuleItem()`
  
          Constructor for an empty RuleItem

`RuleItem(ItemSet premise,
ItemSet consequence,
int genTime,
int ruleSupport,
double[] m_midPoints,
java.util.Hashtable m_priors)`
  
          Constructor

`RuleItem(RuleItem toCopy)`
  
          Constructor that generates a RuleItem out of a given one


| **Method Summary** | |
| --- | --- |
| `double` | `accuracy()`             Gets the expected predictive accuracy of a rule |
| `int` | `compareTo(java.lang.Object o)`             compares two RuleItems and allows an ordering concerning expected predictive accuracy and time of generation Note: this class has a natural ordering that is inconsistent with equals |
| `ItemSet` | `consequence()`             Gets the consequence of a rule |
| `boolean` | `equals(java.lang.Object o)`             returns whether two RuleItems are equal |
| `RuleItem` | `generateRuleItem(ItemSet premise, ItemSet consequence, Instances instances, int genTime, int minRuleCount, double[] m_midPoints, java.util.Hashtable m_priors)`             Constructs a new RuleItem if the support of the given rule is above the support threshold. |
| `java.lang.String` | `getRevision()`             Returns the revision string. |
| `ItemSet` | `premise()`             Gets the premise of a rule |

**Methods inherited from class java.lang.Object**

`getClass, hashCode, notify, notifyAll, toString, wait, wait, wait`

**Constructor Detail**

### RuleItem

```
public RuleItem()
```

:   Constructor for an empty RuleItem

---


### RuleItem

```
public RuleItem(RuleItem toCopy)
```

:   Constructor that generates a RuleItem out of a given one

    **Parameters:**: `toCopy` - RuleItem to copy

---


### RuleItem

```
public RuleItem(ItemSet premise,
                ItemSet consequence,
                int genTime,
                int ruleSupport,
                double[] m_midPoints,
                java.util.Hashtable m_priors)
```

:   Constructor

    **Parameters:**: `premise` - the premise of the future RuleItem: `consequence` - the consequence of the future RuleItem: `genTime` - the time of generation of the future RuleItem: `ruleSupport` - support of the rule: `m_midPoints` - the mid poitns of the intervals: `m_priors` - Hashtable containing the estimated prior probablilities


**Method Detail**

### generateRuleItem

```
public RuleItem generateRuleItem(ItemSet premise,
                                 ItemSet consequence,
                                 Instances instances,
                                 int genTime,
                                 int minRuleCount,
                                 double[] m_midPoints,
                                 java.util.Hashtable m_priors)
```

:   Constructs a new RuleItem if the support of the given rule is above the support threshold.

    :   **Parameters:**: `premise` - the premise: `consequence` - the consequence: `instances` - the instances: `genTime` - the time of generation of the current premise and consequence: `minRuleCount` - the support threshold: `m_midPoints` - the mid points of the intervals: `m_priors` - the estimated priori probabilities (in a hashtable) **Returns:**: a RuleItem if its support is above the threshold, null otherwise

---


### compareTo

```
public int compareTo(java.lang.Object o)
```

:   compares two RuleItems and allows an ordering concerning
    expected predictive accuracy and time of generation
    Note: this class has a natural ordering that is inconsistent with equals

    :   **Specified by:**: `compareTo` in interface `java.lang.Comparable`
    :   **Parameters:**: `o` - RuleItem to compare **Returns:**: integer indicating the sort oder of the two RuleItems

---


### equals

```
public boolean equals(java.lang.Object o)
```

:   returns whether two RuleItems are equal

    :   **Overrides:**: `equals` in class `java.lang.Object`
    :   **Parameters:**: `o` - RuleItem to compare **Returns:**: true if the rules are equal, false otherwise

---


### accuracy

```
public double accuracy()
```

:   Gets the expected predictive accuracy of a rule

    :   **Returns:**: the expected predictive accuracy of a rule stored as a RuleItem

---


### premise

```
public ItemSet premise()
```

:   Gets the premise of a rule

    :   **Returns:**: the premise of a rule stored as a RuleItem

---


### consequence

```
public ItemSet consequence()
```

:   Gets the consequence of a rule

    :   **Returns:**: the consequence of a rule stored as a RuleItem

---


### getRevision

```
public java.lang.String getRevision()
```

:   Returns the revision string.

    :   **Specified by:**: `getRevision` in interface `RevisionHandler`
    :   **Returns:**: the revision


---


|  |  |  |
| --- | --- | --- |
| **Overview** \| **Package** \| **Class** \| **Tree** \| **Deprecated** \| **Index** \| **Help** \| **Weka's home** | |  |
| **PREV CLASS**   **NEXT CLASS** | **FRAMES**    **NO FRAMES**     **All Classes** |  |
| SUMMARY: NESTED \| FIELD \| CONSTR \| METHOD | DETAIL: FIELD \| CONSTR \| METHOD |  |


---
